# Lack of reliability of nanotechnology in the of free plasma DNA in samples of patients with prostate cancer

**DOI:** 10.1186/1755-7682-6-2

**Published:** 2013-01-12

**Authors:** Ricardo Moreno, Pamela O Delgado, Patrícia G Coelho, Sarah R Marsicano, Viviane AV Boas, Ligia A Azzalis, Virgínia BC Junqueira, Katya C Rocha, Luiz Carlos de Abreu, Vitor E Valenti, Jefferson Drezzet, Edimar Cristiano Pereira, Fernando LA Fonseca

**Affiliations:** 1 Laboratório de Análises Clínicas, Faculdade de Medicina do ABC, Av. Príncipe de Gales 821, Santo André, CEP: 09060-650, Brazil; 2 Departamento de Ciências Biológicas, Universidade Federal de São Paulo, UNIFESP, Rua Prof. Artur Riedel, 275, Diadema, São Paulo 09972-270, Brazil; 3 Laboratório de Delineamento de Estudos e Escrita Científica, Departamento de Morfologia e Fisiologia, Faculdade de Medicina do ABC, Santo André, Av. Príncipe de Gales, n. 821, Santo André, CEP: 09060-650, Brazil; 4 Programa de Pós-Graduação em Fisioterapia, Faculdade de Ciências e Tecnologia, Universidade Estadual Paulista, UNESP, Rua Roberto Simonsen, 305, Presidente Prudente, São Paulo 19060-900, Brazil

**Keywords:** Nanotechnology, DNA, Neoplams, Prostate

## Abstract

**Background:**

Several studies seek biological markers that give diagnostic and degree of tumor development. The aim of this study was to validate the determination of plasma DNA using nanotechnology (Nanovue™-NV) in samples of 80 patients with prostate cancer.

**Methods:**

Blood samples of 80 patients of the Urology Ambulatory of Faculdade de Medicina do ABC with prostate cancer confirmed by anatomical-pathology criteria were analyzed. DNA extraction was performed using a GFX TM kit (Amersham Pharmacia Biotech, Inc, USA) following the adapted protocol. Plasma was subjected to centrifugation.

**Results:**

There was a big difference between the first and the second value obtained by NanoVue Only two samples had no differences between duplicates. Maximum difference between duplicates was 38 μg/mL. Average variation between 51 samples was 10.29 μg/mL, although 21 samples had differences above this average. No correlation was observed between pDNA obtained by traditional spectrophotometry and by nanotechnology.

**Conclusion:**

Determination of plasma DNA by nanotechnology was not reproducible.

## Introduction

A tumor is usually used as a synonym for a neoplasm impairment that may or may not be formed by an abnormal growth of neoplastic cells that appears enlarged in extent. Tumor is not synonymous with cancer. While cancer is by definition malignant, a tumor can be benign, pre-malignant, or malignant, or can represent a lesion without any cancerous potential whatsoever [1-3]. Prostate Cancer (PC) or prostatic adenocarcinoma is a malignancy affecting thousands of people worldwide. PC is the sixth most common cancer and the most prevalent in men [4].

Prostate Specific Antigen (PSA) has a good sensibility but a low specificity. Benign prostatic hyperplasia, prostatitis and sexual activity may also increase its serum level. Therefore, PSA is an organ-specific marker, but not a cancer-specific marker. Digital Rectal Examination (DRE) is also used to diagnose PC, but its sensibility depends on the expertise and experience of the examiner [5].

Screening with PSA has been shown to be partially effective, since the number of PC diagnoses increased. On the other hand, screening with PSA and/or DRE were not associated with mortality decreased in patients with PC [6,7].

Patients suffering from diseases in which there are increase in cell death have higher amounts of plasmatic DNA (pDNA) compared with healthy subjects [8,9]. This phenomenon has been observed in patients diagnosed with cancer in which pDNA displays several genetic changes, such as mutations of p53 and Ras genes. Lyses of circulating tumor cells in the blood could also explain this increase [10].

As described above, pDNA is a valuable marker to diseases with genetic changes, such as cancer and could provide an assay for diagnosis and prognosis as well as evaluation of therapeutic interventions. The aim of this study was to validate the determination of plasma DNA using nanotechnology (Nanovue™-NV) in samples of patients with prostate cancer.

## Methods

Blood samples of 80 patients of the Urology Ambulatory of Faculdade de Medicina do ABC with prostate cancer confirmed by anatomical-pathology criteria were analyzed. This study was approved by Ethic and Research Committee of Faculdade de Medicina do ABC, after that about 15 mL of peripheral blood were collected from the patients to determine plasma DNA. EDTA blood was centrifuged at 1,300 g for 10 min. Plasma was transferred into polypropylene tubes and microcentrifuged at 2,400 g.

DNA extraction was performed using a GFX TM kit (Amersham Pharmacia Biotech, Inc, USA) following the adapted protocol. Plasma was subjected to centrifugation. In 1 mL of sample were added 500 μL of extraction solution and the mixture was incubated at room temperature for 10 minutes with occasional agitation. This mixture was eluted five times by the same column of the kit, and after multiple elutions, material was centrifuged at 8,000 g for one minute and the remainder present in the collection tube was discarded. 500 μL of washing solution was added to wash the column and it was centrifuged at 14,000 g for three minutes and eluate present in the collection tube was discarded to clear the column of interferences and improve the quality of DNA to be eluted in the next step. DNA elution consisted of the addition of 20 μL of MiliQ water at 70°C on the column and it was centrifuged at 8,000 g for one minute.

DNA concentration was determined by spectrophotometry (GeneQuant RNA/DNA Calculator Spectrophotometer - Amersham Pharmacia Biotech Biochrom Ltd., USA) or nanotechnology (NanoVue™-NV - General Eletrics Healthcare Limited, UK) measuring absorbance of the samples at 260 – 280 nm.

All statistical analyzes were made using the statistical software package SPSS (v16.0; SPSS, Chicago, IL), and MedCalc software. Statistical significance was considered at p < 0.05.

## Results and discussion

Although, plasma DNA assessment had been made in duplicate to ensure reliability, there was a big difference between the first and the second value obtained by NanoVue (Table 1). In order to avoid technical differences, pipetting were done always by the same person.

**Table 1:** Plasmatic DNA (pDNA) assessed by nanotechnology and respective differences

***SAMPLE***		***[ ] μg/mL***		***SAMPLE***		***[ ] μg/mL***	
**NUMBER**	**[ ] pDNA 1**	**[ ] pDNA 2**	**DIFFERENCE**	**NUMBER**	**[ ] pDNA 1**	**[ ] pDNA 2**	**DIFFERENCE**
**1**	59	51	***8***	**27**	42.5	31	***11.5***
**2**	130.5	92.5	***38***	**28**	50	47	***3***
**3**	37	36	***1***	**29**	51.5	50.5	***1***
**4**	100.5	76	***24.5***	**30**	132.5	132.5	***0***
**5**	26	25.5	***0.5***	**31**	65.5	56.5	***9***
**6**	129.5	133.5	***4***	**32**	84	101	***17***
**7**	142.5	146.5	***4***	**33**	161.5	150	***11.5***
**8**	74	59	***15***	**34**	37.5	20	***17.5***
**9**	59.5	41	***18.5***	**35**	97.5	82.5	***15***
**10**	70.5	63	***7.5***	**36**	121.5	150.5	***29***
**11**	52.5	43.5	***9***	**37**	55	52	***3***
**12**	71.5	64.5	***7***	**38**	136.5	131	***5.5***
**13**	67	65.5	***1.5***	**39**	128.5	102.5	***26***
**14**	88.5	72	***16.5***	**40**	36.5	28	***8.5***
**15**	85	78.5	***6.5***	**41**	90.5	72.5	***18***
**16**	98.5	88.5	***10***	**42**	109.9	105	***4.9***
**17**	49.5	48.5	***1***	**43**	197.5	197	***0.5***
**18**	128.5	154	***25.5***	**44**	133.5	130	***3.5***
**19**	61.5	49.5	***12***	**45**	46	45.5	***0.5***
**20**	80	59	***21***	**46**	56	49	***7***
**21**	71	68.5	***2.5***	**47**	71.5	57.5	***14***
**22**	140.5	140.5	***0***	**48**	80	79	***1***
**23**	159.5	167.5	***8***	**49**	99	75	***24***
**24**	30	24.5	***5.5***	**50**	116.5	134.5	***18***
**25**	72	60	***12***	**51**	47	45.5	***1.5***
**26**	187.5	172	***15.5***				

No correlation was observed between pDNA obtained by traditional spectrophotometry and by nanotechnology (Table 2).

**Table 2:** Lack of correlation between pDNA assessed by GENEQUANT and by NANOVUE

***SAMPLE***	***GENEQUANT***	***Average NANOVUE***	***SAMPLE***	***GENEQUANT***	***Average NANOVUE***
**NUMBER**	**[ ] pDNA**^**1**^	**[ ] pDNA**^**1,2**^	**NUMBER**	**[ ] pDNA**^**1**^	**[ ] pDNA**^**1,2**^
**1**	0.2	55	**27**	4.2	36.75
**2**	0.25	111.5	**28**	4.93	48.5
**3**	0.33	36.5	**29**	5.6	51
**4**	0.6	88.25	**30**	5.73	132.5
**5**	0.63	25.75	**31**	6.5	61
**6**	0.63	131.5	**32**	6.77	92.5
**7**	0.67	144.5	**33**	6.9	155.75
**8**	0.76	66.5	**34**	7.13	28.75
**9**	1	50.25	**35**	7.27	90
**10**	1.2	66.75	**36**	7.8	136
**11**	1.3	48	**37**	7.87	53.5
**12**	1.3	68	**38**	7.87	133.75
**13**	1.3	66.25	**39**	7.97	115.5
**14**	1.4	80.25	**40**	8.87	32.25
**15**	1.47	81.75	**41**	10.5	81.5
**16**	1.6	93.5	**42**	11.2	107.45
**17**	1.7	49	**43**	11.3	197.25
**18**	2	141.25	**44**	11.6	131.75
**19**	2.1	55.5	**45**	12.3	45.75
**20**	2.1	69.5	**46**	13.2	52.5
**21**	2.1	69.75	**47**	15.8	64.5
**22**	2.2	140.5	**48**	17.37	79.5
**23**	3.03	163.5	**49**	17.8	87
**24**	3.37	27.25	**50**	20.7	125.5
**25**	3.37	66	**51**	23	46.25
**26**	4.17	179.75			

^1^ μg/mL; ^2^Average between duplicates.

**Figure 1:**
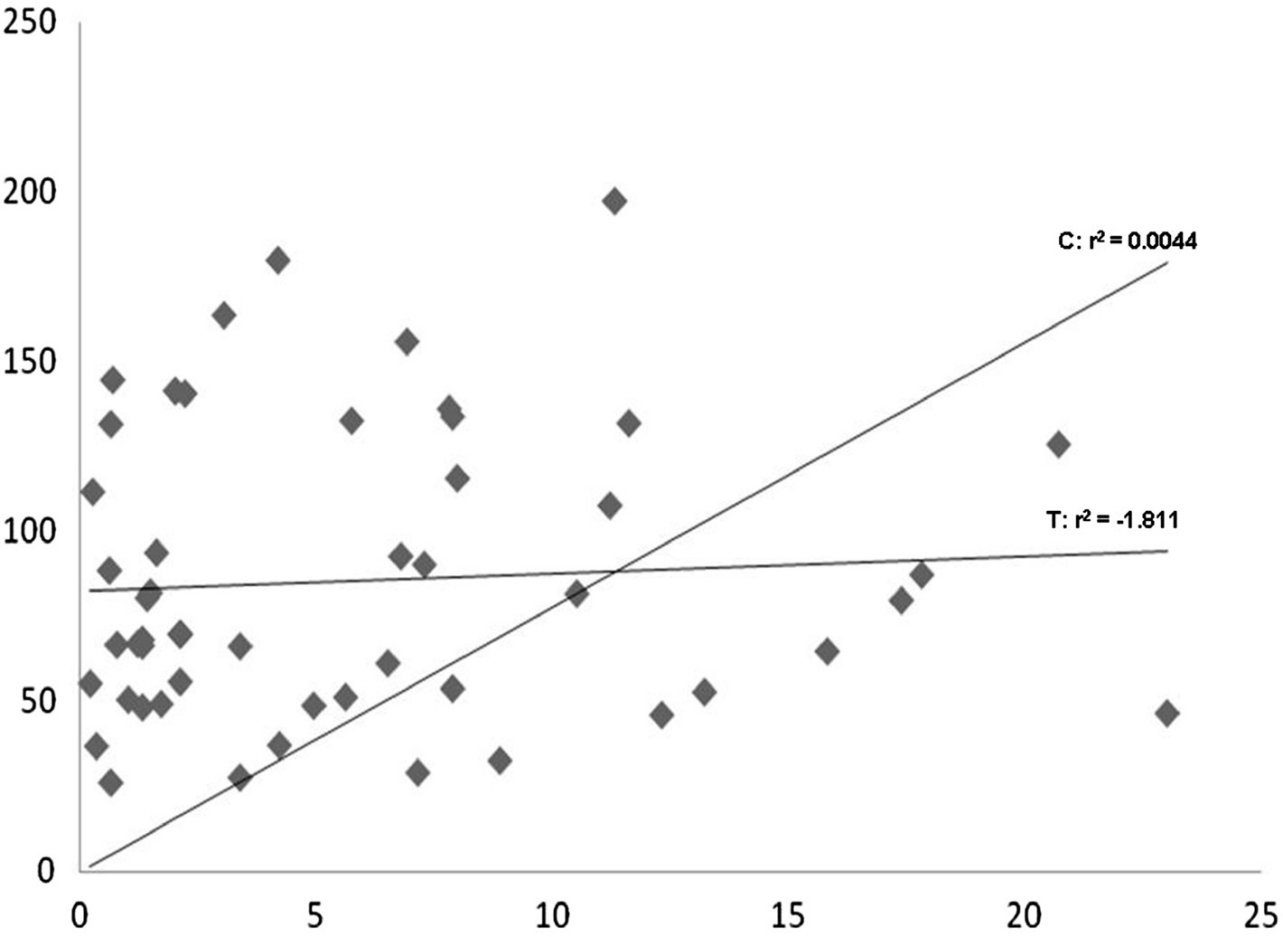
Correlation between the two methods.

In order to compare pDNA obtained by both methods, data were analyzed in a dispersion graph. Result after graph analyzes has shown that r2 correlation was 0.0044 (curve C), indicating no correlation between two methods. Moreover, tendency line shows that results did not have congruity, since r2 obtained was - 1,811 (curve T) (Figure 1).

Plasma DNA was performed in duplicates. 51 samples (63.75%) from the total of 80 were analyzed by nanotechnology when determinations were stopped due to total disagreement between duplicates and lack of reliability. As a main result, the present study indicated that determination of plasma DNA by nanotechnology was not reproducible in patients with prostate cancer. pDNA detection in the circulation has been considered as a promising and non invasive test-diagnosis, as well as a predictor of recurrence or therapeutic answer [11-14].The problem has been being the occurrence of false-positive, especially in patients with autoimmune/inflammatory diseases or recent history of trauma/surgery [10,15].

Based on our data, we found no association between pDNA obtained by traditional spectrophotometry and those obtained by nanotechnology. Absence of correlation could be occurred because values obtained by traditional technique were different from those assessed by nanotechnology. For example, pDNA in sample 22 was 2,2 μg/mL by traditional technique versus 140,5 μg/mL by nanotechnology. In sample 30, value was 5.73 μg/mL by GeneQuant versus 132.5 μg/mL by NanoVue. In fact, elevated pDNA levels are observed in patients with malignant neoplasia, like prostate cancer when compared with healthy individuals [16,17]. Several studies correlate pDNA detection as segment for patients with PC, however most of them using Real-Time PCR technique [18,19]. There are few reports in literature concerning pDNA detection by nanotechnology in patients with PC.

We also reported that only two samples (0.04%) presented no differences between duplicates (samples 22 and 30). Maximum difference between duplicates was 38 μg/mL (sample 2). Average variation between 51 samples was 10.29 μg/mL, although 21 samples (41.2) had differences above this average. Recent evidence has shown elevated levels of cell-free plasma DNA in cancer patients. Allen and coworkers [18] compared and quantified the levels of cell-free pDNA in prostate cancer patients, prostatic intraepithelial neoplasia patients, and benign prostatic hypertrophy patients in order to verify whether it offered a useful diagnostic test. As a main conclusion, the authors indicated that quantification of cell-free pDNA may present a relevant diagnostic function in distinguishing malignant and benign prostatic disorders. In the same context, Boddy and colleagues [19] assessed the potential of cell-free DNA levels as a prostate cancer diagnostic indicator. The study reported that high levels are present in plasma samples of patients with prostate cancer compared with healthy subjects, however, it can not be considered for diagnostic value during the management of prostate cancer.

These data present relevant information, since currently pDNA detection in the circulation has been considered as a promising and non invasive test-diagnosis [20,21] and for the public health system violation [22]. Therefore, these findings open new perspectives for more research and may benefit experimental and clinical investigations.

## Conclusion

This study showed that determination of plasma DNA by nanotechnology was not reproducible in patients with prostate cancer.

## Competing interests

The authors declare that they have no competing interests.

## Author’s contributions

RM, POD, PGC, SRM, VAVB, LAA, VBCJ, KCR, LCdeA, VEV, ECP, JD and FLAF participated in the revision of the manuscript. FLAF, LCdeA, VEV determined the design, interpreted the text and drafted the manuscript. All authors read and gave final approval for the version submitted for publication.
